# Choroid Plexus Enlargement and USPIO‐Based Inflammatory Feature in Cerebral Small Vessel Disease

**DOI:** 10.1002/acn3.70382

**Published:** 2026-04-05

**Authors:** Yongqiang Qu, Xiaoning Ren, Cuihua Fan, Chun Li, Lijie Zhang, Zaiqiang Zhang, Shaowu Li, Zhongmeng Lai, Bin Cai, Ying Fu, Yi Lin, Zhibao Zhu

**Affiliations:** ^1^ Department of Neurology and Institute of Neurology of First Affiliated Hospital Institute of Neuroscience, and Fujian Key Laboratory of Molecular Neurology, Fujian Medical University Fuzhou China; ^2^ Department of Anesthesiology Fujian Medical University Union Hospital Fuzhou China; ^3^ Department of Blood Transfusion The First Affiliated Hospital of Fujian Medical University Fuzhou China; ^4^ Department of Neurology National Regional Medical Center, Binhai Campus of the First Affiliated Hospital, Fujian Medical University Fuzhou China; ^5^ Department of Neurology Beijing Tiantan Hospital, Capital Medical University Beijing China; ^6^ Department of Radiology Beijing Tiantan Hospital, Capital Medical University Beijing China

**Keywords:** choroid plexus, CSVD, inflammation, MRI, USPIO

## Abstract

**Objective:**

The choroid plexus (CP) is a key component of the blood–cerebrospinal fluid barrier (BCSFB), but its mechanism of action in cerebral small vessel disease (CSVD) remains unclear. This study investigated CP volume (CPV) alterations and their association with conventional imaging markers in CSVD and explored the underlying role of inflammation using ultrasmall superparamagnetic particles of iron oxide (USPIO)‐enhanced MRI.

**Methods:**

In this study, 111 CSVD patients and 69 healthy controls (HC) underwent 3.0 T conventional MRI to quantify CPV in the lateral (LV), third (3 V), and fourth (4 V) ventricles. CSVD burden was evaluated using visual assessment scales, including indicators such as white matter hyperintensities (WMH), lacunes, basal ganglia‐enlarged perivascular spaces (BG‐EPVS), cerebral microbleeds (CMBs), and brain atrophy (BA). A subgroup (22 CSVD, 10 HC) underwent USPIO‐enhanced MRI to measure the change of signal intensity ratio (∆SIR) as a feature of inflammatory activity in the CP and deep gray matter nuclei.

**Results:**

CSVD patients showed significantly larger CPV (all *p* < 0.001). CPV demonstrated significant positive correlations with the severity of conventional CSVD imaging markers (all *p* < 0.05). USPIO‐enhanced MRI revealed an increased ΔSIR in the LV, 3 V, and 4 V CP (all *p* < 0.05). Furthermore, increased USPIO uptake was observed in deep gray matter nuclei in CSVD. Crucially, a positive correlation was found between LV CP volume and ΔSIR (*r* = 0.375, *p* = 0.034), and the LV CP inflammation was strongly correlated with the hippocampus (*r* = 0.582, *p* < 0.001).

**Interpretation:**

CP enlargement is prominent in CSVD and closely linked to the CSVD imaging burden. CP inflammation is associated with increased volume. CP may be a novel biomarker to help diagnose, monitor, and treat neuroinflammation in CSVD.

**Abbreviations**
3 V: Third ventricle
4 V: Fourth ventricle
AC: Anterior cingulate
AD: Alzheimer's disease
ANOVA: Analysis of variance
AT: Anterior temporal
BA: Brain atrophy
BBB: Blood–brain barrier
BCSFB: Blood‐cerebrospinal fluid barrier
BG‐EPVS: Basal ganglia‐enlarged perivascular spaces
CMBs: Cerebral microbleeds
CNh: Caudate nucleus head
CP: Choroid plexus
CPV: Choroid plexus volume
CSF: Cerebrospinal fluid
CSVD: Cerebral small vessel disease
FI: Fronto‐insula
GPe: Globus pallidus externus
HC: Healthy controls
Hip: Hippocampus
ICC: Intraclass correlation coefficient
IQR: Inter‐quartile range
LV: Lateral ventricle
MO: Medulla oblongata
MRI: Magnetic resonance imaging
MS: Multiple sclerosis
MTA: Medial temporal atrophy
OF: Orbito‐frontal
Put: Putamen
ROI: Region of interest
SD: Standard deviation
SI: Signal intensity
SIR: Signal intensity ratio
Tha: Thalamus
TIV: Total intracranial volume
USPIO: Ultrasmall superparamagnetic particles of iron oxide
WMH: White matter hyperintensity

## 1 Introduction

Cerebral small vessel disease (CSVD) refers to a group of disorders affecting the small blood vessels in the brain [1]. Typical pathological markers of CSVD include white matter hyperintensities (WMH), presumed vasogenic lacunar infarcts, enlarged perivascular spaces (EPVS), cerebral microbleeds (CMBs), and brain atrophy (BA) [2]. CSVD is common in the aging process and is a major cause of stroke, cognitive impairment, and functional decline [1, 2]. Although the association between glymphatic dysfunction and dementia has been widely recognized [3], few studies have explored its role in CSVD.

The glymphatic pathway acts as a brain clearance system, mainly by pushing cerebrospinal fluid (CSF) into the brain tissue and removing metabolic waste [4, 5]. In this process, the choroid plexus (CP) plays a crucial role in regulating CSF dynamics and maintaining homeostasis within the brain [6, 7, 8]. Enlargement of the CP has been reported in neurodegenerative and neuroinflammatory diseases [9], and age‐related CSF changes are associated with structural remodeling of the CP [10]. Considering the widespread coexistence of CSVD and Alzheimer's disease (AD) in the elderly [11, 12], CP in CSVD may represent a previously overlooked but important neuroimaging biomarker.

The CP contains multiple immune cells and expresses various inflammatory mediators [13], serving as a key site for neuroinflammation [14]. As research advances, neuroinflammation has increasingly been recognized as a key mechanism in the progression of CSVD [15, 16]. However, quantifying neuroinflammation in vivo has been challenging. Ultrasmall superparamagnetic particles of iron oxide (USPIO) can be absorbed by macrophages and microglia, leading to their accumulation in inflammatory regions [17], which provides a tool for in vivo monitoring of inflammatory processes. This technology has been successfully used in Multiple sclerosis (MS) [18] and stroke [19, 20] research, but its application in CSVD, especially for assessing CP inflammation, remains mostly unexplored.

There is an association between increased CP volume (CPV) and inflammation [21], but it is unclear if this occurs in CSVD. Using conventional and USPIO‐enhanced magnetic resonance imaging (MRI), this study investigates CP structural and inflammatory changes in CSVD, which may provide crucial insights into understanding the pathogenesis of CSVD.

## 2 Methods

### 2.1 Study Design and Subjects

The CSVD participants and healthy controls (HC) were prospectively recruited from 2016 to 2021. The Regional Ethics Review Committee of the First Affiliated Hospital of Fujian Medical University and Tiantan Hospital of Capital Medical University approved the study protocol and informed consent procedures. All individuals provided written informed consent to participate in this study.

CSVD was diagnosed through clinical symptoms and MRI imaging markers [2, 22], and HC had no neurological or systemic disease history. Patients who do not fall within these categories, including those exhibiting MRI findings of CSVD combined with other brain pathologies (such as acute cerebral infarction), incomplete MRI sequences and data, or individuals under 18 years of age, were explicitly excluded.

### 2.2 MRI Acquisition

The study used a 3.0 T MRI scanner (Siemens Healthcare, Erlangen, Germany) with a 64‐channel head coil. All participants underwent brain conventional MRI, and the MRI scan parameters: (1) A 3D T1‐weighted sequence (TR = 2300 ms, TE = 2.32 ms, FlipAngle = 8°, FOV = 240 × 240 mm^2^, slice thickness = 0.9 mm, number of slices = 192); (2) A T2‐weighted sequence (TR = 5000 ms, TE = 105 ms, FlipAngle = 150°, FOV = 199 × 220 mm^2^, slice thickness = 3 mm, number of slices = 33); (3) A T2‐weighted fluid‐attenuated inversion recovery (FLAIR) sequence (TR = 9000 ms, TE = 81 ms, TI = 2500 ms, FlipAngle = 150°, FOV = 220 × 220 mm^2^, slice thickness = 5 mm, number of slices = 20); (4) A susceptibility‐weighted imaging (SWI) sequence (TR = 29 ms, TE = 20 ms, FlipAngle = 15°, FOV = 192 × 220 mm^2^, slice thickness = 1.5 mm, number of slices = 48). A subgroup was scanned at baseline to determine the presence of any acute cerebrovascular lesions (pre‐USPIO scan). If none were found, a USPIO (ferumoxytol; AMAG Pharmaceuticals, Cambridge, MA; 3 mg/kg) infusion was performed immediately, followed by a second scan 48 h later (post‐USPIO scan). The USPIO‐enhanced MRI scan parameters: A 3D T1‐weighted sequence (TR = 810 ms, TE = 10 ms, FlipAngle = 120°, FOV = 199 × 199 mm^2^, slice thickness = 0.7 mm, number of slices = 176).

### 2.3 Image Analysis

#### 2.3.1 Quality Control of Image Assessment

All image assessments relying on visual evaluation or manual segmentation were independently completed by two assessors with more than 3 years of experience in neuroimaging analysis, who remained blinded to clinical information and group assignments. The assessors received standardized training and used the same assessment guidelines and software tools. Assessors first independently scored or segmented all biomarkers. After completion, inter‐assessor reliability was calculated using internal consistency tests. If inconsistencies arose, a consensus‐building phase was initiated in which both assessors jointly reviewed the images and reached agreement. If consensus was still not reached, a third senior imaging expert served as the arbitrator and made the final decision. This process significantly improved the consistency and reproducibility of manual assessments.

#### 2.3.2 Conventional CSVD Imaging Markers Assessment

Two experienced neuroradiologists (Z.‐B.Z. and Y.F.) visually assessed imaging markers according to the Standards for Reporting Vascular Changes on Neuroimaging (STRIVE) guidelines [2, 23]. We used a visual assessment by combining the T1, T2, T2 FLAIR, and SWI images to evaluate WMH, lacunes, basal ganglia‐EPVS (BG‐EPVS), CMBs, and BA. The total CSVD score, ranging from 0 to 6, was determined based on individual imaging markers [24]. The BA score is based on six visual assessment scales, including orbito‐frontal (OF), anterior cingulate (AC), anterior temporal (AT), fronto‐insula (FI), medial temporal atrophy (MTA), and posterior atrophy (PA) [25]. Detailed visual assessment procedures and consistency tests are provided in Tables S1–S4.

#### 2.3.3 Choroid Plexus Volume Segmentation

**FIGURE 1:**
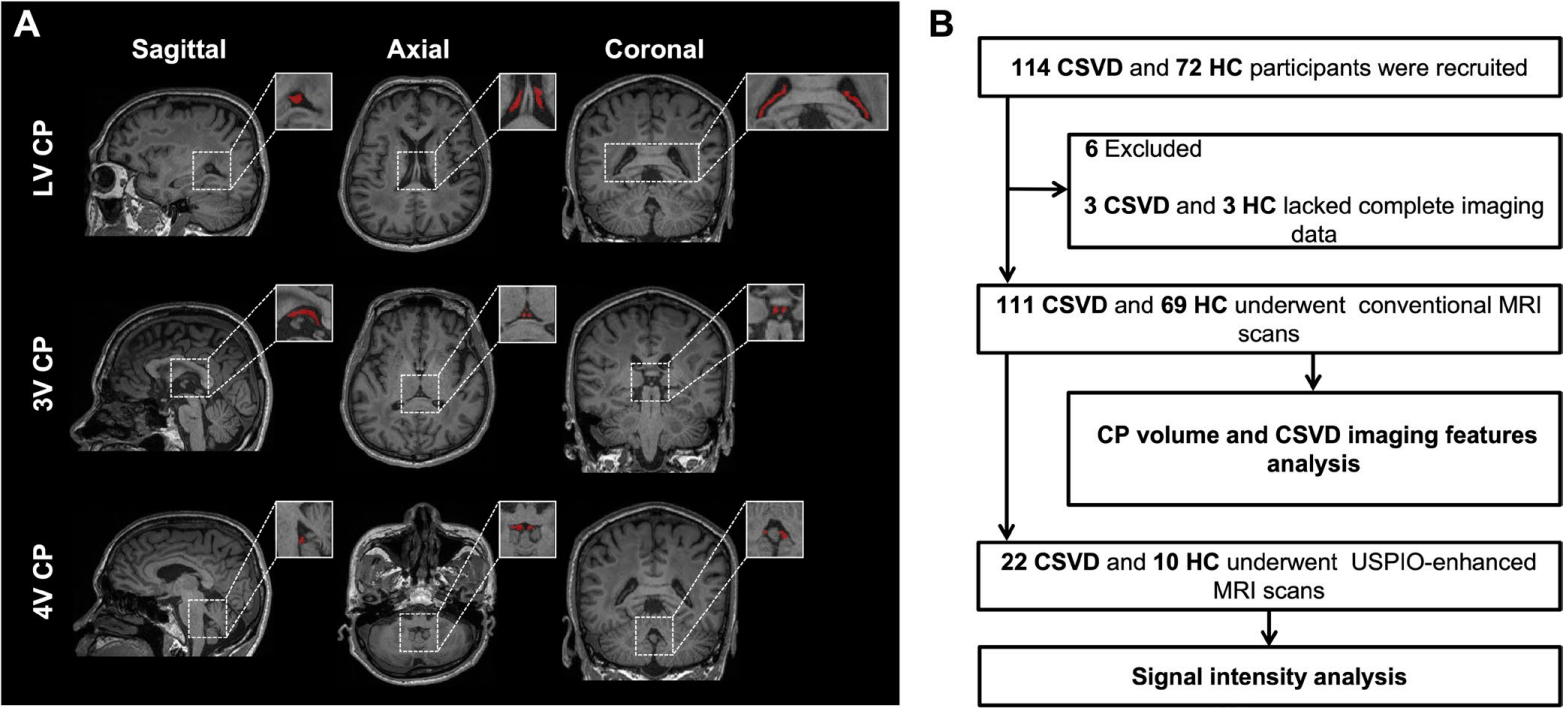
Choroid plexus volume segmentation and participant flow chart. (A) Representative images showing manual segmentation of the LV, 3 V, and 4 V CP (highlighted in red) across sagittal, axial, and coronal views on 3D T1‐weighted MRI. (B) Flowchart of study participants. 3 V, third ventricle; 4 V, fourth ventricle; CP, choroid plexus; CSVD, cerebral small vessel disease; HC, healthy controls; LV, lateral ventricle; USPIO, ultrasmall superparamagnetic particles of iron oxide.

Using ITK‐SNAP software version 4.0 (www.itksnap.org), two trained physicians (Y.‐Q.Q. and X.‐N.R.) independently performed manual segmentation of the CPV [26]. Figure 1A shows the region of interest (ROI), which includes the lateral ventricle (LV), the third ventricle (3 V), and the fourth ventricle (4 V). The total intracranial volume (TIV) was measured for normalization using the SPM toolbox CAT12 (https://www.neuro.uni‐jena.de/cat/). The final result is expressed as CPV corrected for the TIV (ratio of TIV × 10^3^). The LV CPV from both hemispheres showed no significant differences initially. Therefore, to simplify the model and enhance statistical power, we ultimately used a combined CPV. Related explanations have been added, and the results of the hemispheric analysis are provided in Figure S1. The intraclass correlation coefficient (ICC) for CP segmentation agreement was excellent (ICC = 0.895; 95% CI, 0.748–0.948; *p* < 0.001).

#### 2.3.4 Inflammation Measurement

Previous studies have shown that the measurement of USPIO uptake can be quantified by using relative MRI signal intensity (SI) [27, 28], and the signal intensity ratio (SIR) was defined as the ratio of the ROI to the mean signal intensity of neck muscle tissue [29]. The ROI for inflammation assessment includes CP in different ventricles (LV, 3 V, and 4 V), and deep gray matter nuclei, including hippocampus (Hip), caudate nucleus head (CNh), putamen (Put), globus pallidus externus (GPe), thalamus (Tha), pons, and medulla oblongata (MO). Two trained physicians (Y.‐Q.Q. and X.‐N.R.) also used ITK‐SNAP software to independently manually segment the ROI and calculate the SIR. Our inflammation measurements also extracted data from both hemispheres separately, and preliminary analysis revealed no significant hemispheric differences (Figures S4 and S5). Ultimately, we decided to use the average values of the bilateral SIR. The relevant results and the consistency of inflammation measurements are detailed in Table S5. Finally, quantitative analysis methods were employed to calculate the change of SIR (∆SIR) before and after USPIO administration: ∆SIR = SIRpost—SIRpre. Where SIRpre and SIRpost represent SIR before and after USPIO injection, respectively. Higher ΔSIR values indicate more significant USPIO uptake, suggesting more active inflammation.

### 2.4 Statistical Analysis

Statistical analyses were performed using IBM SPSS Statistics version 26.0, R 4.5.0, and Prism 9.5.0. Continuous variables with normal distribution are presented as mean ± standard deviation (SD) and analyzed using independent samples *t*‐tests or analysis of variance (ANOVA). Non‐normally distributed data are presented as medians alongside their inter‐quartile range (IQR) and analyzed using the nonparametric test. The Chi‐square test (*χ* ^2^) is used for gender. To evaluate the associations between CP ∆SIR and other variables (including CPV, conventional CSVD imaging markers, and deep gray matter nuclei ∆SIR), Pearson correlation coefficients were calculated. Furthermore, partial correlation models were implemented to control for age and sex, ensuring that the observed associations were independent of these potential confounders. Cohen's kappa tests were used to evaluate the interobserver agreement in the degree of conventional CSVD imaging markers and BA score. ICC was used to evaluate the agreement of CPV segmentation and inflammation measurement. All tests of significance were 2‐tailed, and *p* < 0.05 was considered statistically significant.

## 3 Results

### 3.1 Study Population Characteristics

The inclusion criteria for the research participants are shown in Figure 1B. A total of 111 patients with CSVD and 69 HC were included in the conventional MRI analysis. A subgroup of 22 CSVD patients and 10 HC underwent USPIO‐enhanced MRI. As shown in Table 1, the CSVD group was significantly older than HC (53.65 ± 13.63 vs. 39.38 ± 14.23 years, *p* < 0.001), but there were no significant differences in gender distribution (*p* = 0.402). In the USPIO subgroup, CSVD and HC were included for comparison by matching age (51.73 ± 13.42 vs. 50.00 ± 16.04 years, *p* = 0.753) and gender (*p* = 0.631).

**TABLE 1:** Demographic characteristics and choroid plexus volume of the study population.

	Conventional MRI		*p*	USPIO‐enhanced MRI		*p*
	CSVD *N* = 111	HC *N* = 69		CSVD *N* = 22	HC *N* = 10	
Age, year, mean (± SD)	53.65 (± 13.63)	39.38 (± 14.23)	< 0.001	51.73 (± 13.42)	50.00 (± 16.04)	0.753
Female, *N* (%)	54 (48.65)	38 (55.07)	0.402	9 (40.91)	5 (50.00)	0.631
LV CPV (ratio of TIV × 10^3^), median (IQR)	1.74 (1.47, 2.06)	1.51 (1.30, 1.73)	< 0.001	1.74 (1.43, 2.10)	1.41 (1.25, 1.49)	0.005
3 V CPV (ratio of TIV × 10^3^), median (IQR)	0.34 (0.27, 0.45)	0.20 (0.15, 0.25)	< 0.001	0.39 (0.30, 0.54)	0.17 (0.13, 0.19)	< 0.001
4 V CPV (ratio of TIV × 10^3^), median (IQR)	0.17 (0.13, 0.23)	0.12 (0.09, 0.16)	< 0.001	0.26 (0.21, 0.31)	0.13 (0.11, 0.14)	< 0.001

*Note:* Normally distributed data are presented as mean (± SD); non‐normally distributed data are presented as median (IQR); age using an independent *t*‐test; the Chi‐square test (*χ* ^2^) is used for gender; CPV group comparisons are made using the nonparametric test; *p* < 0.05 was considered statistically significant. Abbreviations: 3 V, third ventricle; 4 V, fourth ventricle; CPV, choroid plexus volume; CSVD, cerebral small vessel disease; HC, healthy control; IQR, inter‐quartile ranges; V, lateral ventricle; MRI, magnetic resonance imaging; *N*, number; SD, standard deviation; TIV, total intracranial volume; USPIO, ultrasmall superparamagnetic particles of iron oxide.

### 3.2 Comparison of Choroid Plexus Volume

The analysis of CPV (normalized to TIV) confirmed widespread enlargement of CP in CSVD. Crucially, LV CPV was the largest, followed by 3 V and 4 V. In the main group, the LV, 3 V, and 4 V CPV were significantly larger in CSVD patients compared with HC. As detailed in Table 1, the median LV CPV was 1.74 (IQR: 1.47, 2.06) in CSVD vs. 1.51 (IQR: 1.30, 1.73) in HC (*p* < 0.001). Similarly, 3 V CPV was 0.34 (IQR: 0.27, 0.45) in CSVD vs. 0.20 (IQR: 0.15, 0.25) in HC (*p* < 0.001), and 4 V CPV was 0.17 (IQR: 0.13, 0.23) in CSVD vs. 0.12 (IQR: 0.09, 0.16) in HC (*p* < 0.001). The difference remained significant after adjusting for age and sex (all *p* < 0.001).

This pattern was consistent in the USPIO subgroup. Median LV CPV was 1.74 (IQR: 1.43, 2.10) in CSVD vs. 1.41 (IQR: 1.25, 1.49) in HC (*p* = 0.005), 3 V CPV was 0.39 (IQR: 0.30, 0.54) in CSVD vs. 0.17 (IQR: 0.13, 0.19) in HC (*p* < 0.001), and 4 V CPV was 0.26 (IQR: 0.21, 0.31) in CSVD vs. 0.13 (IQR: 0.11, 0.14) in HC (*p* < 0.001) (Table 1).

### 3.3 Relationship Between Choroid Plexus Volume and Conventional CSVD Imaging Markers

Spearman correlations indicate significant positive relationships between larger CPV and the severity of conventional CSVD imaging markers, suggesting a potential structural biomarker for disease burden (Table 2). The LV CPV showed significant correlations with WMH score (ρ = 0.230, *p* = 0.015), lacunes score (ρ = 0.219, *p* = 0.021), BG‐EPVS score (ρ = 0.320, *p* = 0.001), CMBs score (ρ = 0.213, *p* = 0.025), and the total CSVD score (ρ = 0.333, *p* < 0.001). 3 V CPV and 4 V CPV were significantly correlated with WMH, lacunes, CMBs, and the total CSVD score (all *p* < 0.05), but not with BG‐EPVS (3 V, *p* = 0.416; 4 V, *p* = 0.479). These findings were visually supported by Figure S2. Furthermore, the LV, 3 V, and 4 V CPV were significantly correlated with the total BA score (all *p* < 0.001), with specific patterns of association with regional atrophy scores (e.g., OF, AC) detailed in Figure S3 and Table S6.

**TABLE 2:** Correlation between choroid plexus volume and conventional CSVD imaging markers in CSVD.

		LV CPV (ratio of TIV × 10^3^)	3 V CPV (ratio of TIV × 10^3^)	4 V CPV (ratio of TIV × 10^3^)
WMH score	ρ	0.230	0.349	0.360
	*p*	0.015	< 0.001	< 0.001
Lacunes score	ρ	0.219	0.236	0.266
	*p*	0.021	0.013	0.005
BG‐EPVS score	ρ	0.320	0.078	0.068
	*p*	0.001	0.416	0.479
CMBs score	ρ	0.213	0.359	0.290
	*p*	0.025	< 0.001	0.002
Total CSVD score	ρ	0.333	0.380	0.360
	*p*	< 0.001	< 0.001	< 0.001
Total BA score	ρ	0.349	0.372	0.328
	*p*	< 0.001	< 0.001	< 0.001

*Note:* The relationship was analyzed using Spearman correlation; *p* < 0.05 was considered statistically significant. Abbreviations: 3 V, third ventricle; 4 V, fourth ventricle; BA, brain atrophy; G‐EPVS, basal ganglia‐enlarged perivascular spaces; CMBs, cerebral microbleeds; CPV, choroid plexus volume; CSVD, cerebral small vessel disease; LV, lateral ventricle; TIV, total intracranial volume; WMH, white matter hyperintensity.

### 3.4 Inflammatory Feature of Choroid Plexus and Deep Gray Matter Nuclei

**FIGURE 2:**
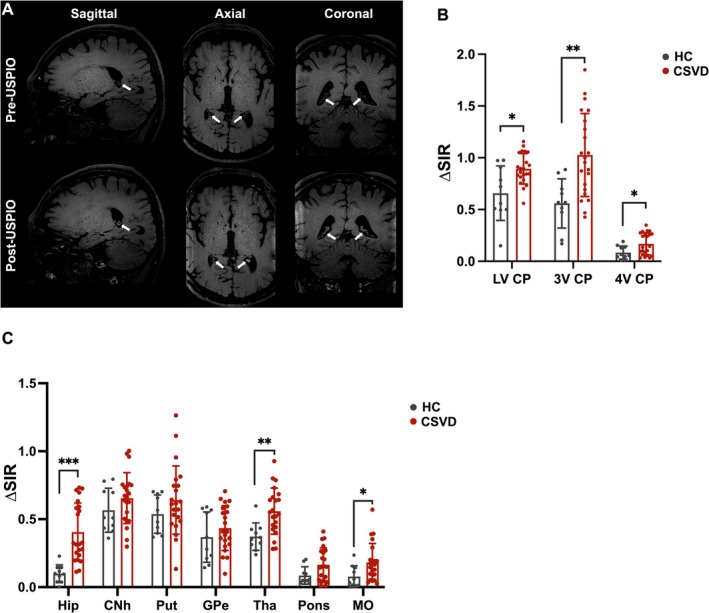
Inflammatory feature of the choroid plexus and deep gray matter nuclei on USPIO‐enhanced MRI. (A) Representative images showing the LV CP (white arrow) across sagittal, axial, and coronal views on 3D T1‐weighted MRI. There is no signal change on the pre‐USPIO 3D‐T1 images. However, after the USPIO injection 48 h, the LV CP shows hyperintensity on the post‐USPIO 3D‐T1 images. (B) Comparison of the ΔSIR (indicating USPIO uptake) in the LV, 3 V, and 4 V CP between CSVD patients and HC. CSVD showed a significantly higher ΔSIR, suggesting prominent inflammatory activity. (C) Quantitative analysis of the ΔSIR in deep gray matter nuclei. CSVD patients exhibited significantly increased USPIO uptake in the Hip, Tha, and MO regions, indicating widespread neuroinflammation. Data are presented as mean ± SD; group comparisons used an independent *t*‐test. *p < 0.05; **p < 0.01;***p < 0.001. 3 V, third ventricle; 4 V, fourth ventricle; CNh, caudate nucleus head; CP, choroid plexus; CSVD, cerebral small vessel disease; GPe, globus pallidus externus; HC, healthy controls; Hip, hippocampus; LV, lateral ventricle; MO, medulla oblongata; Put, putamen; SD, standard deviation; Tha, thalamus; USPIO, ultrasmall superparamagnetic particles of iron oxide; SIR, signal intensity ratio.

USPIO‐enhanced MRI highlighted active inflammation in CSVD. Representative images show clear hyperintensity in the LV CP after USPIO administration in Figure 2A. Quantitatively, the ∆SIR was significantly higher in the LV, 3 V, and 4 V CP of CSVD patients compared with HC (Figure 2B, all *p* < 0.05). Moreover, CSVD patients exhibited significantly increased USPIO uptake in deep gray matter nuclei, including the regions of Hip, Tha, and MO (Figure 2C, all *p* < 0.05), reflecting widespread neuroinflammation beyond deep gray matter nuclei. Figures S6 and S7 further confirmed these findings, showing significant post‐USPIO signal enhancement in CSVD compared with HC across all CP regions and specific deep gray matter nuclei.

### 3.5 Relationship Between Inflammatory Feature and Conventional CSVD Imaging Markers

Pearson correlation analysis examined the relationship between inflammation (∆SIR) and the severity of conventional CSVD imaging markers (Table S7). LV CP ∆SIR showed a significant positive correlation with WMH score (*r* = 0.509, *p* = 0.015). 4 V CP ∆SIR was significantly correlated with WMH score (*r* = 0.578, *p* = 0.005) and lacunes score (*r* = 0.458, *p* = 0.032). Additionally, ∆SIR in several deep gray matter nuclei, including the Put and Tha, was positively correlated with WMH score (*r* = 0.469, *p* = 0.028, and *r* = 0.474, *p* = 0.026, respectively). These findings suggest that CP inflammation might be involved in the neuroinflammatory process associated with small vessel damage.

### 3.6 Connection Between Choroid Plexus Volume and Inflammatory Feature

**FIGURE 3:**
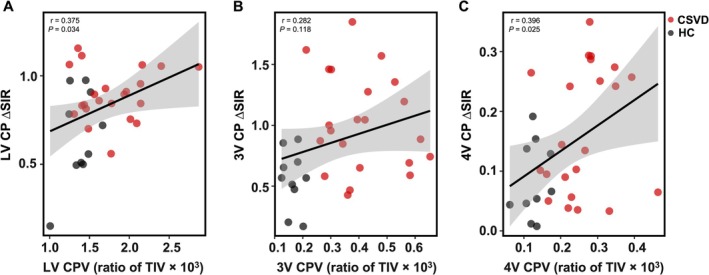
The connection between choroid plexus volume and inflammation. (A) The scatter plot shows a positive correlation (*r* = 0.375, *p* = 0.034) between LV CPV and CP inflammation (measured by USPIO‐induced ΔSIR). This suggests that CP enlargement is associated with neuroinflammation. (B) In 3 V CP, no significant correlation was observed (*r* = 0.282, *p* = 0.118). (C) However, a significant correlation was found in 4 V CP (*r* = 0.396, *p* = 0.025). The relationship was analyzed using Pearson correlation. *p* < 0.05 was considered statistically significant. 3 V, third ventricle; 4 V, fourth ventricle; CP, choroid plexus; CPV, choroid plexus volume; CSVD, cerebral small vessel disease; HC, healthy controls; LV, lateral ventricle; SIR, signal intensity ratio; TIV, total intracranial volume; USPIO, ultrasmall superparamagnetic particles of iron oxide.

A key finding was the significant positive correlation between CPV and its local inflammatory activity. As shown in Figure 3, LV CPV was positively correlated with its own ∆SIR (*r* = 0.375, *p* = 0.034) and, more strongly, with the ∆SIR of the 4 V CP (*r* = 0.396, *p* = 0.025). No significant correlation was found for the 3 V CP (*r* = 0.282, *p* = 0.118). The correlation results remained consistent after adjusting for age and sex in a partial correlation model (LV CP, *r* = 0.406, *p* = 0.026; 3 V CP, *r* = 0.278, *p* = 0.137; 4 V CP, *r* = 0.403, *p* = 0.027). Furthermore, a strong network of correlations was observed, with inflammation in the LV CP significantly linked to the Hip (*r* = 0.582, *p* < 0.001), Tha (*r* = 0.640, *p* < 0.001), and other subcortical structures (Table 3). These findings remained consistent after adjusting for age and sex using partial correlation analysis (Table S8). The above results indicate that CP enlargement is closely linked to neuroinflammation, supporting the hypothesis that CP may contribute to glymphatic dysfunction and deep gray matter inflammation in CSVD.

**TABLE 3:** Correlation of inflammation between choroid plexus and deep gray matter nuclei.

		LV CP ∆SIR	3 V CP ∆SIR	4 V CP ∆SIR
Hip ∆SIR	*r*	0.582	0.424	0.614
	*p*	< 0.001	0.016	< 0.001
CNh ∆SIR	*r*	0.431	0.089	0.304
	*p*	0.014	0.629	0.091
Put ∆SIR	*r*	0.372	0.155	0.371
	*p*	0.036	0.397	0.036
GPe ∆SIR	*r*	0.421	−0.017	0.465
	*p*	0.016	0.926	0.007
Tha ∆SIR	*r*	0.640	0.303	0.477
	*p*	< 0.001	0.091	0.006
Pons ∆SIR	*r*	0.362	0.367	0.484
	*p*	0.042	0.039	0.005
MO ∆SIR	*r*	0.314	0.466	0.553
	*p*	0.081	0.007	0.001

*Note:* The relationship was analyzed using Pearson correlation; *P* < 0.05 was considered statistically significant. Abbreviations: 3 V, third ventricle; 4 V, fourth ventricle; CNh, caudate nucleus head; CP, choroid plexus; GPe, globus pallidus externus; Hip, hippocampus; LV, lateral ventricle; MO, medulla oblongata; Put, putamen; SIR, signal intensity ratio; Tha, thalamus.

## 4 Discussion

This research provides several novel insights into the contribution of the CP in CSVD. We found that CP enlargement is a pervasive feature of CSVD, correlating with the severity of conventional CSVD imaging markers. USPIO‐enhanced MRI offers direct in vivo evidence of CP active inflammation in CSVD. This neuroinflammatory feature extends beyond the CP to affect deep gray matter nuclei essential for cognitive function. Finally, significant structure–function relationships exist between CPV enlargement and CP local inflammation. In overview, it provides a new perspective on understanding the neuroinflammatory mechanisms of CSVD, suggesting that the CP may play a key role in CSVD pathophysiology.

Our study strongly confirms that CP enlargement is a hallmark of CSVD. The consistent rise in the LV, 3 V, and 4 V CPV indicates a widespread process impacting the entire blood‐CSF barrier (BCSFB) system, rather than a localized event [13]. It aligns with the findings of several recent studies [12, 30, 31], showing that CPV enlargement may be a common feature in CSVD. More importantly, we included a more comprehensive panel of CSVD markers and found a significant positive correlation between CPV and CSVD burden, particularly showing the strongest correlation with WMH. One possible explanation is that CP dysfunction might alter CSF, impacting the glymphatic system and worsening white matter damage [32]. Furthermore, the CPV correlates with BA scores, indicating a potential role in neurodegenerative processes of CSVD [33].

The CP frequently exhibits volume, permeability, and immune activity alterations in neurodegenerative and inflammatory diseases [10, 34, 35, 36, 37], which provides pathological and biological support for the USPIO outcome observed in CSVD. The most significant contribution of our work is the direct demonstration of CP inflammation using USPIO‐enhanced MRI. USPIO‐enhanced MRI signals can be affected by blood retention, perfusion, and blood–brain barrier (BBB) permeability, especially early after infusion [20, 38]. However, pharmacokinetic results indicate that USPIO particles are primarily cleared by the spleen and liver, with plasma levels falling below detection within 24–48 h [17, 39]. Therefore, we performed an MRI 48 h after USPIO injection to minimize hemodynamic interference. The signal changes primarily reflect iron uptake by activated myeloid cells (e.g., macrophages, microglia), enabling precise targeting of neuroinflammation [19, 40].

The significantly higher CP inflammation (∆SIR) provides tangible evidence of active phagocytosis by myeloid cells, offering a long‐sought in vivo validation of the neuroinflammatory hypothesis in CSVD [15]. The extension of significant USPIO uptake to deep gray matter nuclei, particularly the Hip and Tha, reveals a spreading inflammatory process. Previous work focused on the BBB breakdown as an early and widespread biomarker of cognitive impairment [41]. However, our study found that the strong correlations of LV CP enlargement and inflammation with Hip inflammation indicate a potential BCSFB mechanism linking CSVD to AD.

The positive correlation between volume and ∆SIR in the CP is a key finding, indicating that CP structural enlargement is not merely a passive change, but inflammation may actively promote the process. The structure–function relationship offers a hypothesis to explain the role of CP in CSVD: chronic cerebral hypoperfusion or endothelial dysfunction in CSVD triggers an inflammatory response in the highly vascularized CP [42]. This causes immune cell infiltration and cellular hypertrophy, resulting in increased structural remodeling volume. Moreover, inflamed CP may also release pro‐inflammatory cytokines and alter CSF composition, leading to BCSFB disruption and reducing waste clearance efficiency [32, 33, 43]. It creates a vicious cycle of neuroinflammation and brain injury, which further promotes neurodegeneration [16, 44, 45].

Our findings have immediate clinical and translational implications. First, CPV may serve as a novel imaging biomarker for CSVD, complementing existing assessment tools. Second, interventions targeting the CP‐CSF system could emerge as potential therapeutic strategies for CSVD. Finally, combined with USPIO‐enhanced MRI technology, noninvasive monitoring of neuroinflammation in CSVD patients can provide an objective index for evaluating the efficacy of anti‐inflammatory treatments. Future longitudinal studies should explore whether CP enlargement and inflammation occur before the appearance of WMH and other CSVD imaging markers, which would confirm the early diagnostic value of CP. Combining USPIO‐MRI with CSF biomarker analysis could further elucidate the molecular pathways involved.

This study has several limitations. The cross‐sectional design prevents causal inference. We find that the unweighted summation of imaging markers is clinically practical for assessing CSVD burden; it overlooks differences in pathological importance and interdependence among features, potentially biasing interpretation. While USPIO enhancement suggests inflammatory responses, histological validation is lacking. Future studies combining dynamic contrast‐enhanced MRI with different contrast agents could further elucidate the contributions of these complementary vascular and inflammatory processes. Our USPIO subgroup, while providing important proof‐of‐concept, was small, requiring validation in larger cohorts. CP function indicators, such as CSF production rate and composition changes, were not assessed.

In conclusion, our study uses multimodal neuroimaging MRI to identify the CP as crucial in CSVD. USPIO enhancement shows CP inflammation linked to volume increase and a broader brain inflammatory response. Targeting the CP could help diagnose, monitor, and treat CSVD neuroinflammation.

## Author Contributions

Z.‐B.Z., Y.F. formulated the study concept and designed the study. Y.F., Y.L., S.‐W.L. Z.‐Q.Z., B.C., and Z.‐M.L. recruited the patients. Z.‐B.Z., Y.‐Q.Q., X.‐N.R., C.‐H.F., C.L., and L.‐J.Z. collected and analyzed data. Y.‐Q.Q. and X.‐N.R. wrote the manuscript.

## Funding

This work has been supported by the grants U21A20360 (Y.F.), 82371349 (Y.F.), U22A20296 (Y.L.) from the National Natural Science Foundation of China, the grants 2022ZQNZD005 (Y.F.) from the Major Scientific Research Program for Young and Middle‐aged Health Professionals of Fujian Province, and the grants 2021Y9131 (Y.L.) from the Joint Funds for the Innovation of Science and Technology of Fujian Province.

## Ethics Statement

This study received approval from the Regional Ethics Review Committee of the First Affiliated Hospital of Fujian Medical University and the Tiantan Hospital of Capital Medical University. Participant information was collected only after patients and HC understood the study and gave written informed consent.

## Consent

All authors gave their consent for publication.

## Conflicts of Interest

The authors declare no conflicts of interest.

## Supporting information


**Figure S1:** Comparison of the left and right hemispheres of LV CPV. No statistically significant difference was observed in LV CPV between the left and right hemispheres (*p* = 0.526). The Wilcoxon signed‐rank test was used to assess LV CPV differences between the left and right hemispheres. CPV, choroid plexus volume; LV, lateral ventricle; TIV, total intracranial volume.
**Figure S2:** Relationship of CPV with WMH, lacunes, BG‐EPVS, CMBs, and total CSVD score in CSVD (a–c). An increased LV, 3 V, and 4 V CPV in CSVD patients is associated with greater severity of WMH (d–f). Larger LV, 3 V, and 4 V CPV in CSVD are associated with a higher chance of developing lacunes (g–i). Higher LV CPV in CSVD correlates with more severe BG‐EPVS, but not 3 V and 4 V CPV (j–l). CSVD patients have larger LV, 3 V, and 4 V CPV, and more CMBs (m–o). The larger LV and 3 V CPV in CSVD link to a higher total CSVD score, but 4 V CPV is not. Data are presented as median; group comparisons used the nonparametric test. *p < 0.05; **p < 0.01; ***p < 0.001. 3 V, third ventricle; 4 V, fourth ventricle; BG‐EPVS, basal ganglia‐enlarged perivascular spaces; CMBs, cerebral microbleeds; CPV, choroid plexus volume; IQR, inter‐quartile range; LV, lateral ventricle; TIV, total intracranial volume; WMH, white matter hyperintensity.
**Figure S3:** Relationship between CPV and BA score in CSVD (a–c). An increased LV, 3 V, and 4 V CPV in CSVD patients is associated with OF score (d–f). Larger LV CPV in CSVD is linked to increased AC score, but not 3 V and 4 V CPV (g–i). The LV, 3 V, and 4 V CPV in CSVD do not correlate with AT score (j–l). CSVD patients have larger 3 V CPV with a higher FI score, while there are no observations in LV and 4 V CPV (m–o). The larger LV, 3 V, and 4 V CPV in CSVD are not linked to a change in MTA score (p–r). The larger LV, 3 V, and 4 V CPV in CSVD don't seem to affect the PA score. Data are presented as median; group comparisons used the nonparametric test. *p < 0.05; **p < 0.01. 3 V, third ventricle; 4 V, fourth ventricle; AC, anterior cingulate; AT, anterior temporal; CPV, choroid plexus volume; FI, fronto‐insula; IQR, inter‐quartile range; LV, lateral ventricle; MTA, medial temporal atrophy; OF, orbito‐frontal; PA, posterior atrophy; TIV, total intracranial volume;
**Figure S4:** Comparison of the left and right hemispheres of pre‐USPIO SIR. There was no difference of pre‐USPIO SIR between the left and right hemispheres in (a) the LV CP (*p* = 0.658), (b) Hip (*p* = 0.354), (c) CNh (*p* = 0.515), (d) GPe (*p* = 0.729), (e) Put (*p* = 0.734), and (f) Tha (*p* = 0.452). Hemispheric comparisons were performed using paired Student's *t*‐tests. CP, choroid plexus; CNh, caudate nucleus head; GPe, globus pallidus externus; Hip, hippocampus; LV, lateral ventricle; Put, putamen; Tha, thalamus; SIR, signal intensity ratio; USPIO, ultrasmall superparamagnetic particles of iron oxide.
**Figure S5:** Comparison of the left and right hemispheres of post‐USPIO SIR. There was no difference of post‐USPIO SIR between the left and right hemispheres in (a) the LV CP (*p* = 0.767), (b) Hip (*p* = 0.923), (c) CNh (*p* = 0.672), (d) GPe (*p* = 0.940), (e) Put (*p* = 0.801), and (f) Tha (*p* = 0.625). Hemispheric comparisons were performed using paired Student's *t*‐tests. CP, choroid plexus; CNh, caudate nucleus head; Hip, hippocampus; GPe, globus pallidus externus; LV, lateral ventricle; Put, putamen; SIR, signal intensity ratio; Tha, thalamus; USPIO, ultrasmall superparamagnetic particles of iron oxide.
**Figure S6:** Signal Intensity Ratio of Choroid Plexus in CSVD and HC (a–c). Comparing signal intensity changes pre‐ and post‐USPIO, significant signal enhancement was observed in both HC and CSVD at LV, 3 V, and 4 V CP (d–e). In pre‐USPIO, there was no significant SIR difference between HC and CSVD at LV, 3 V, and 4 V CP. Post‐USPIO, CSVD had a significantly higher SIR than HC, showing a notable difference. Data are presented as mean ± SD; group comparisons used an independent *t*‐test; *p < 0.05; **p < 0.01; ***p < 0.001; ****p < 0.0001. 3 V, third ventricle; 4 V, fourth ventricle; CP, choroid plexus; CSVD, cerebral small vessel disease; HC, healthy controls; LV, lateral ventricle; SD, standard deviation; SIR, signal intensity ratio; USPIO, ultrasmall superparamagnetic particles of iron oxide.
**Figure S7:** Signal Intensity Ratio of Deep Gray Matter Nuclei in CSVD and HC (a–g). Comparing signal intensity changes pre‐ and post‐USPIO, significant signal enhancement was seen in both HC and CSVD at deep gray matter nuclei, including Hip, CNh, Put, GPe, Tha, pons, and MO (h–i). In pre‐USPIO, no significant SIR difference existed between HC and CSVD at deep gray matter nuclei. Post‐USPIO, CSVD showed a much higher SIR than HC in the Hip, Tha, and MO, highlighting a clear difference. Data are presented as mean ± SD; group comparisons used an independent *t*‐test; *p < 0.05; **p < 0.01; *** *p* < 0.001; ****p < 0.0001. CNh, caudate nucleus head; CSVD, cerebral small vessel disease; HC, healthy controls; Hip, hippocampus; GPe, globus pallidus externus; MO, medulla oblongata; Put, putamen; Tha, thalamus; SD, standard deviation; SIR, signal intensity ratio; USPIO, ultrasmall superparamagnetic particles of iron oxide.
**Table S1:** Definition of visual assessment of conventional CSVD imaging markers.
**Table S2:** The method for calculating the total CSVD score.
**Table S3:** Brain atrophy visual rating protocol.
**Table S4:** Cohen's kappa tests for conventional CSVD imaging markers and BA score agreement.
**Table S5:** Intraclass correlation coefficient for gray matter nuclei SIR agreement.
**Table S6:** The association between choroid plexus volume and conventional CSVD imaging markers in CSVD.
**Table S7:** Correlation between inflammation and conventional CSVD imaging markers in CSVD.
**Table S8:** Correlation of inflammation between choroid plexus and deep gray matter nuclei.

## Data Availability

The data that support the findings of this study are available from the corresponding author upon reasonable request.
