# Supplementary material for: Choroid Plexus Enlargement and USPIO‐Based Inflammatory Feature in Cerebral Small Vessel Disease
Source: Ann Clin Transl Neurol. 2026 Apr 5;13(10):2078–87. doi: 10.1002/acn3.70382 (PMC13394647; doi:10.1002/acn3.70382)
Supplement: Supplementary file 1 [file ACN3-13-2078-s001.docx]

**Supplementary materials**

**Figure S1.** Comparison of the left and right hemispheres of LV CPV.

**Figure S2.** Relationship of CPV with WMH, lacunes, BG-EPVS, CMBs, and total CSVD score in CSVD.

**Figure S3.** Relationship between CPV and BA score in CSVD.

**Figure S4.** Comparison of the left and right hemispheres of pre-USPIO SIR.

**Figure S5.** Comparison of the left and right hemispheres of post-USPIO SIR.

**Figure S6.** Signal Intensity Ratio of Choroid Plexus in CSVD and HC.

**Figure S7.** Signal Intensity Ratio of Deep Gray Matter Nuclei in CSVD and HC.

**Table S1.** Definition of visual assessment of conventional CSVD imaging markers.

**Table S2.** The method for calculating the total CSVD score.

**Table S3.** Brain atrophy visual rating protocol.

**Table S4.** Cohen’s kappa tests for conventional CSVD imaging markers and BA score agreement.

**Table S5.** Intraclass correlation coefficient for gray matter nuclei SIR agreement.

**Table S6.** The association between choroid plexus volume and conventional CSVD imaging markers in CSVD.

**Table S7.** Correlation between inflammation and conventional CSVD imaging markers in CSVD.

**Table S8.** Correlation of inflammation between choroid plexus and deep gray matter nuclei.

**Supplementary Figure Legends**

**Figure S1. Comparison of the left and right hemispheres of LV CPV.** No statistically significant difference was observed in LV CPV between the left and right hemispheres (*P* = 0.526). The Wilcoxon signed-rank test was used to assess LV CPV differences between the left and right hemispheres. Abbreviations: CPV, choroid plexus volume; LV, lateral ventricle; TIV, total intracranial volume.

**Figure S2. Relationship of CPV with WMH, lacunes, BG-EPVS, CMBs, and total CSVD score in CSVD.** (a-c) An increased LV, 3V, and 4V CPV in CSVD patients is associated with greater severity of WMH. (d-f) Larger LV, 3V, and 4V CPV in CSVD are associated with a higher chance of developing lacunes. (g-i) Higher LV CPV in CSVD correlates with more severe BG-EPVS, but not 3V and 4V CPV. (j-l) CSVD patients have larger LV, 3V, and 4V CPV, and more CMBs. (m-o) The larger LV and 3V CPV in CSVD link to a higher total CSVD score, but 4V CPV is not. Data are presented as median; group comparisons used the nonparametric test. * *P* < 0.05; ** *P* < 0.01; *** *P* < 0.001. Abbreviations: LV, lateral ventricle; 3V, third ventricle; 4V, fourth ventricle; CPV, choroid plexus volume; TIV, total intracranial volume; WMH, white matter hyperintensity; BG-EPVS, basal ganglia-enlarged perivascular spaces; CMBs, cerebral microbleeds; IQR, inter-quartile range.

**Figure S3. Relationship between CPV and BA score in CSVD.** (a-c) An increased LV, 3V, and 4V CPV in CSVD patients is associated with OF score. (d-f) Larger LV CPV in CSVD is linked to increased AC score, but not 3V and 4V CPV. (g-i) The LV, 3V, and 4V CPV in CSVD do not correlate with AT score. (j-l) CSVD patients have larger 3V CPV with a higher FI score, while there are no observations in LV and 4V CPV. (m-o) The larger LV, 3V, and 4V CPV in CSVD are not linked to a change in MTA score. (p-r) The larger LV, 3V, and 4V CPV in CSVD don’t seem to affect the PA score. Data are presented as median; group comparisons used the nonparametric test. * *P* < 0.05; ** *P* < 0.01. Abbreviations: LV, lateral ventricle; 3V, third ventricle; 4V, fourth ventricle; CPV, choroid plexus volume; TIV, total intracranial volume; OF, orbito-frontal; AC, anterior cingulate; AT, anterior temporal; FI, fronto-insula; MTA, medial temporal atrophy; PA, posterior atrophy; IQR, inter-quartile range.

**Figure S4. Comparison of the left and right hemispheres of pre-USPIO SIR.** There was no difference of pre-USPIO SIR between the left and right hemispheres in (a) the LV CP (*P* = 0.658), (b) Hip (*P* = 0.354), (c) CNh (*P* = 0.515), (d) GPe (*P* = 0.729), (e) Put (*P* = 0.734), and (f) Tha (*P* = 0.452). Hemispheric comparisons were performed using paired Student’s *t*-tests. Abbreviations: USPIO, ultrasmall superparamagnetic particles of iron oxide; SIR, signal intensity ratio; LV, lateral ventricle; CP, choroid plexus; Hip, hippocampus; CNh, caudate nucleus head; GPe, globus pallidus externus; Put, putamen; Tha, thalamus.

**Figure S5. Comparison of the left and right hemispheres of post-USPIO SIR.** There was no difference of post-USPIO SIR between the left and right hemispheres in (a) the LV CP (*P* = 0.767), (b) Hip (*P* = 0.923), (c) CNh (*P* = 0.672), (d) GPe (*P* = 0.940), (e) Put (*P* = 0.801), and (f) Tha (*P* = 0.625). Hemispheric comparisons were performed using paired Student’s *t*-tests. Abbreviations: USPIO, ultrasmall superparamagnetic particles of iron oxide; SIR, signal intensity ratio; LV, lateral ventricle; CP, choroid plexus; Hip, hippocampus; CNh, caudate nucleus head; GPe, globus pallidus externus; Put, putamen; Tha, thalamus.

**Figure S6. Signal Intensity Ratio of Choroid Plexus in CSVD and HC.** (a-c) Comparing signal intensity changes pre- and post-USPIO, significant signal enhancement was observed in both HC and CSVD at LV, 3V, and 4V CP. (d-e) In pre-USPIO, there was no significant SIR difference between HC and CSVD at LV, 3V, and 4V CP. Post-USPIO, CSVD had a significantly higher SIR than HC, showing a notable difference. Data are presented as mean ± SD; group comparisons used an independent t-test; * *P* < 0.05; ** *P* < 0.01; *** *P* < 0.001; **** *P* < 0.0001. Abbreviations: USPIO, ultrasmall superparamagnetic particles of iron oxide; CP, choroid plexus; LV, lateral ventricle; 3V, third ventricle; 4V, fourth ventricle; SIR, signal intensity ratio; CSVD, cerebral small vessel disease; HC, healthy controls; SD, standard deviation.

**Figure S7. Signal Intensity Ratio of Deep Gray Matter Nuclei in CSVD and HC.** (a-g) Comparing signal intensity changes pre- and post-USPIO, significant signal enhancement was seen in both HC and CSVD at deep gray matter nuclei, including Hip, CNh, Put, GPe, Tha, pons, and MO. (h-i) In pre-USPIO, no significant SIR difference existed between HC and CSVD at deep gray matter nuclei. Post-USPIO, CSVD showed a much higher SIR than HC in the Hip, Tha, and MO, highlighting a clear difference. Data are presented as mean ± SD; group comparisons used an independent t-test; * *P* < 0.05; ** *P* < 0.01; *** *P* < 0.001; **** *P* < 0.0001. Abbreviations: USPIO, ultrasmall superparamagnetic particles of iron oxide; SIR, signal intensity ratio; CSVD, cerebral small vessel disease; HC, healthy controls; Hip, hippocampus; CNh, caudate nucleus head; GPe, globus pallidus externus; Put, putamen; Tha, thalamus; MO, medulla oblongata; SD, standard deviation.

| **Table S1 Definition of visual assessment of conventional CSVD imaging markers.** | | | |
| --- | --- | --- | --- |
| **Markers** | **Definition** | **Assessment method** | **Detailed Scoring Criteria** |
| **WMH** | Hyperintense on T2/FLAIR, no cavitation, variable size | Fazekas Scale | PWMH (points):  0: Absent  1: Caps or pencil-thin lining  2: Smooth halo  3: Irregular periventricular signal extending into the deep white matter  DWMH (points):  0: Absent  1: Punctate foci  2: Beginning confluence  3: Large confluent areas |
| **Lacunes** | Round/ovoid, subcortical, fluid-filled cavity (3 - 15 mm), CSF-like signal on T2/FLAIR | Count | Absent  Presence (n ≥ 1 lacunes) |
| **BG-EPVS** | < 3 mm, CSF-like signal, follows vessel course | Count EPVS in the slice with the highest number. The numbers refer to EPVS on one side of the brain, and the higher score was used if there was asymmetry between both hemispheres. | Absent or n < 20 BG-EPVS  n ≥ 20 BG-EPVS |
| **CMBs** | Small (generally 2 - 5 mm in diameter, but up to 10 mm), round, signal void with blooming on SWI/T2* | Count | Absent  n = 1-4 microbleeds  n ≥ 5 microbleeds |
| Abbreviations: CSVD, cerebral small vessel disease; WMH, white matter hyperintensity; FLAIR, fluid-attenuated inversion recovery; PWMH, periventricular WMH; DWMH, deep WMH; CSF, cerebrospinal fluid; BG-EPVS, basal ganglia-enlarged perivascular spaces; EPVS, enlarged perivascular spaces; CMBs, cerebral microbleeds; SWI, susceptibility-weighted imaging; n, number. | | | |

| **Table S2 The method for calculating the total CSVD score.** | |
| --- | --- |
| **Markers** | **Total CSVD score (0 - 6 points)** |
| **WMH** | 1: PWMH + DWMH score 3-4 |
|  | 2: PWMH + DWMH WMH score 5-6 |
| **Lacunes** | 1: n ≥ 1 lacunes |
| **BG-EPVS** | 1: n ≥ 20 BG-EPVS |
| **CMBs** | 1: n = 1-4 CMBs |
|  | 2: n ≥ 5 CMBs |
| Abbreviations: CSVD, cerebral small vessel disease; WMH, white matter hyperintensity; PWMH, periventricular WMH; DWMH, deep WMH; BG-EPVS, basal ganglia-enlarged perivascular spaces; CMBs, cerebral microbleeds; n, number. | |

| **Table S3 Brain atrophy visual rating protocol.** | | |
| --- | --- | --- |
| **Region** | **Slice selection** | **Rating guide** |
| **OF** | (a) Corpus callosum not yet visible (pre-rating slice).  (b) Corpus callosum just visible (rate olfactory sulcus and cingulate sulcus on this slice).  (c) Post-rating slice. | 0: Closed sulcus  1: Small sulcal slit, just revealing CSF  2: Opening of the sulcus, CSF clearly visible  3: Severe widening of the sulcus |
| **AC** | (a) Corpus callosum not yet visible (pre-rating slice).  (b) Corpus callosum just visible (rate olfactory sulcus and cingulate sulcus on this slice).  (c) Post-rating slice. | 0: Closed sulcus  1: Sulcal opening (CSF visible), although  narrower towards the peak  2: Sulcal widening along the length of the  sulcus  3: Severe widening of the sulcus |
| **AT** | (a) Connection between the frontal and  temporal lobes is still visible (pre-rating slice).  (b) No visible connection between the  frontal and temporal lobes (rate this slice).  (c) Post-rating slice. | 0: Normal appearances  1: Slight prominence of anterior temporal  sulci  2: Temporal sulci definitely widened  3: Gyri severely atrophic and ribbon-like.  WM and GM cannot be distinguished  (normal temporal lobe at this level is  less substantial than the frontal lobe,  ribbon-like gyri of stage 3 temporal lobe  are similar to stage 4 frontal gyri)  4: Temporal pole has a simple linear profile or is not seen at all |
| **FI** | (a) Anterior commissure (AC) not yet visible (pre-rating slice).  (b) Anterior commisure just visible (rate  this slice and the 2 posterior). | (Average the score over the 3 slices)  0: Closed sulcus  1: Sulcal opening, CSF clearly visible  2: Sulcal widening and the emergence of  an arrow head shape pointing towards  the midline  3: Severe widening along the length of the sulcus |
| **MTA** | (a) In the middle of the hippocampal body, in front of the pons or halfway through the pons depending on the angle of the scan.  (b) Scroll though the hippocampus to get an impression of the atrophy throughout.  (c) Don't rate too close to the amygdala, lf the hippocampus curls up, the slice is too close to the hippocampal head.  (d) At the origin of the fornix, the slice is too close to the tail.  (e) A score of 0 can stil be given if there is some opening of the choroid fissure on a few slices through the hippocampal body if the remainder are closed.  (f) A score of 1 is given if the choroid fissure is opened over the entire length of the hippocampal body. | 0: Normal  1: Widened choroid fissure  2: Increased widening of the choroid fissure, widening of the temporal horn, opening of other sulci (i.e. collateral/fusiform sulcus)  3: Pronounced volume loss of the hippocampus  4: End stage atrophy |
| **PA** | No slice selection, just scroll through parietal lobe, posterior cingulate sulcus, parieto-occipital sulcus, precuneus. | 0: Closed sulci of parietal lobes and cuneus  1: Mild widening of posterior cingulate and parieto-occipital sulci  2: Substantial widening of the sulci  3: Extreme widening of the posterior cingulate and parieto-occipital sulci |
| Abbreviations: OF, orbito-frontal; AC, anterior cingulate; AT, anterior temporal; FI, fronto-insula; MTA, medial temporal atrophy; PA, posterior atrophy. | | |

| **Table S4 Cohen’s kappa tests for conventional CSVD imaging markers and BA score agreement.** | | | |
| --- | --- | --- | --- |
|  | **kappa values** | **95% CI** | ***P*** |
| **WMH score** | 0.837 | 0.808-0.939 | <0.001 |
| **Lacunes score** | 0.838 | 0.737-0.938 | <0.001 |
| **BG-EPVS score** | 0.728 | 0.601-0.856 | <0.001 |
| **CMBs score** | 0.850 | 0.832-0.950 | <0.001 |
| **OF score** | 0.772 | 0.758-0.914 | <0.001 |
| **AC score** | 0.829 | 0.766-0.940 | <0.001 |
| **AT score** | 0.780 | 0.628-0.933 | <0.001 |
| **FI score** | 0.871 | 0.845-0.971 | <0.001 |
| **MTA score** | 0.811 | 0.785-0.946 | <0.001 |
| **PA score** | 0.803 | 0.758-0.941 | <0.001 |
| Abbreviations: CSVD, cerebral small vessel disease; BA, brain atrophy; CI, confidence interval; WMH, white matter hyperintensity; BG-EPVS, basal ganglia-enlarged perivascular spaces; CMBs, cerebral microbleeds; OF, orbito-frontal; AC, anterior cingulate; AT, anterior temporal; FI, fronto-insula; MTA, medial temporal atrophy; PA, posterior atrophy. | | | |

| **Table S5 Intraclass correlation coefficient for inflammation measurements SIR agreement.** | | | |
| --- | --- | --- | --- |
|  | **ICC coefficient** | **95% CI** | ***P*** |
| **LV CP pre-USPIO SIR** | 0.898 | 0.801-0.949 | <0.001 |
| **3V CP pre-USPIO SIR** | 0.902 | 0.809-0.951 | <0.001 |
| **4V CP pre-USPIO SIR** | 0.866 | 0.744-0.932 | <0.001 |
| **Hip pre-USPIO SIR** | 0.811 | 0.649-0.903 | <0.001 |
| **CNh pre-USPIO SIR** | 0.729 | 0.512-0.858 | <0.001 |
| **Put pre-USPIO SIR** | 0.891 | 0.789-0.945 | <0.001 |
| **GPe pre-USPIO SIR** | 0.840 | 0.697-0.919 | <0.001 |
| **Tha pre-USPIO SIR** | 0.789 | 0.610-0.891 | <0.001 |
| **Pons pre-USPIO SIR** | 0.853 | 0.716-0.926 | <0.001 |
| **MO pre-USPIO SIR** | 0.906 | 0.817-0.953 | <0.001 |
| **LV CP post-USPIO SIR** | 0.876 | 0.761-0.938 | <0.001 |
| **3V CP post-USPIO SIR** | 0.883 | 0.773-0.941 | <0.001 |
| **4V CP post-USPIO SIR** | 0.734 | 0.520-0.861 | <0.001 |
| **Hip post-USPIO SIR** | 0.878 | 0.766-0.939 | <0.001 |
| **CNh post-USPIO SIR** | 0.823 | 0.668-0.909 | <0.001 |
| **Put post-USPIO SIR** | 0.751 | 0.549-0.871 | <0.001 |
| **GPe post-USPIO SIR** | 0.855 | 0.724-0.926 | <0.001 |
| **Tha post-USPIO SIR** | 0.746 | 0.540-0.868 | <0.001 |
| **Pons post-USPIO SIR** | 0.848 | 0.711-0.923 | <0.001 |
| **MO post-USPIO SIR** | 0.835 | 0.690-0.916 | <0.001 |
| Abbreviations: SIR, signal intensity ratio; ICC, intraclass correlation coefficient; CI, confidence interval; LV, lateral ventricle; CP, choroid plexus; 3V, third ventricle; 4V, fourth ventricle; Hip, hippocampus; USPIO, ultrasmall superparamagnetic particles of iron oxide; CNh, caudate nucleus head; Put, putamen; GPe, globus pallidus externus; Tha, thalamus; MO, medulla oblongata. | | | |

| **Table S6 The association between choroid plexus volume and conventional CSVD imaging markers in CSVD.** | | | | | | |
| --- | --- | --- | --- | --- | --- | --- |
|  | **LV CPV**  **(ratio of TIV × 10^3^)** | *P* | **3V CPV**  **(ratio of TIV × 10^3^)** | *P* | **4V CPV**  **(ratio of TIV × 10^3^)** | *P* |
| **WMH score** |  | 0.0456 |  | 0.0006 |  | 0.0001 |
| **0** | 1.671 (1.408, 1.941) |  | 0.302 (0.229, 0.373) |  | 0.147 (0.120, 0.194) |  |
| **1** | 1.674 (1.462, 2.048) |  | 0.320 (0.268, 0.412) |  | 0.155 (0.114, 0.213) |  |
| **2** | 1.912 (1.548, 2.437) |  | 0.439 (0.299, 0.546) |  | 0.239 (0.173, 0.280) |  |
| **Lacunes score** |  | 0.0213 |  | 0.0131 |  | 0.0050 |
| **0** | 1.562 (1.406, 1.964) |  | 0.304 (0.250, 0.384) |  | 0.145 (0.110, 0.200) |  |
| **1** | 1.758 (1.520, 2.142) |  | 0.360 (0.280, 0.464) |  | 0.193 (0.138, 0.245) |  |
| **BG-EPVS score** |  | 0.0007 |  | 0.4174 |  | 0.4797 |
| **0** | 1.621 (1.409, 1.963) |  | 0.316 (0.263, 0.446) |  | 0.156 (0.121, 0.242) |  |
| **1** | 1.880 (1.619, 2.293) |  | 0.357 (0.271, 0.461) |  | 0.185 (0.139, 0.224) |  |
| **CMBs score** |  | 0.0009 |  | < 0.0001 |  | 0.0095 |
| **0** | 1.674 (1.470, 2.001) |  | 0.286 (0.235, 0.313) |  | 0.146 (0.114, 0.200) |  |
| **1** | 1.472 (1.381, 1.811) |  | 0.384 (0.307, 0.517) |  | 0.176 (0.118, 0.259) |  |
| **2** | 1.934 (1.718, 2.147) |  | 0.377 (0.292, 0.481) |  | 0.202 (0.139, 0.249) |  |
| **Total CSVD score** |  | 0.0008 |  | 0.0021 |  | 0.0119 |
| **0** | 1.709 (1.479, 1.964) |  | 0.297 (0.250, 0.309) |  | 0.134 (0.122, 0.189) |  |
| **1** | 1.607 (1.316, 2.110) |  | 0.317 (0.220, 0.417) |  | 0.151 (0.109, 0.214) |  |
| **2** | 1.602 (1.450, 1.978) |  | 0.312 (0.237, 0.357) |  | 0.141 (0.108, 0.171) |  |
| **3** | 1.539 (1.394, 1.806) |  | 0.307 (0.253, 0.407) |  | 0.183 (0.121, 0.209) |  |
| **4** | 1.623 (1.403, 2.319) |  | 0.403 (0.306, 0.525) |  | 0.194 (0.123, 0.266) |  |
| **5** | 1.962 (1.701, 2.192) |  | 0.415 (0.343, 0.532) |  | 0.225 (0.158, 0.286) |  |
| **6** | 2.162 (1.747, 2.477) |  | 0.456 (0.278, 0.484) |  | 0.218 (0.170, 0.243) |  |
| **BA score** |  |  |  |  |  |  |
| **OF score** |  | 0.0261 |  | 0.0098 |  | 0.0074 |
| **0** | 1.634 (1.381, 1.932) |  | 0.297 (0.225, 0.385) |  | 0.140 (0.120, 0.186) |  |
| **1** | 1.737 (1.438, 1.935) |  | 0.324 (0.286, 0.433) |  | 0.166 (0.115, 0.230) |  |
| **2** | 1.969 (1.577, 2.293) |  | 0.408 (0.284, 0.461) |  | 0.212 (0.163, 0.257) |  |
| **3** | 1.867 (1.470, 3.521) |  | 0.532 (0.348, 0.742) |  | 0.188 (0.108, 0.321) |  |
| **AC score** |  | 0.0114 |  | 0.0398 |  | 0.1716 |
| **0** | 1.599 (1.402, 1.886) |  | 0.307 (0.260, 0.404) |  | 0.140 (0.120, 0.186) |  |
| **1** | 1.846 (1.520, 2.153) |  | 0.344 (0.271, 0.456) |  | 0.166 (0.115, 0.230) |  |
| **2** | 1.962 (1.582, 2.157) |  | 0.463 (0.313, 0.507) |  | 0.212 (0.163, 0.257) |  |
| **AT score** |  | 0.0690 |  | 0.0397 |  | 0.0215 |
| **0** | 1.590 (1.357, 1.928) |  | 0.273 (0.218, 0.394) |  | 0.143 (0.109, 0.166) |  |
| **1** | 1.739 (1.484, 2.095) |  | 0.343 (0.287, 0.450) |  | 0.186 (0.130, 0.239) |  |
| **2** | 2.062 (1.962, 2.162) |  | 0.437 (0.394, 0.480) |  | 0.275 (0.203, 0.346) |  |
| **FI score** |  | 0.1434 |  | 0.0087 |  | 0.0260 |
| **0** | 1.468 (1.425, 2.080) |  | 0.313 (0.307, 0.456) |  | 0.129 (0.089, 0.156) |  |
| **1** | 1.620 (1.396, 1.934) |  | 0.300 (0.219, 0.370) |  | 0.151 (0.121, 0.201) |  |
| **2** | 1.745 (1.479, 2.141) |  | 0.338 (0.273, 0.455) |  | 0.176 (0.118, 0.227) |  |
| **3** | 1.775 (1.526, 2.156) |  | 0.403 (0.308, 0.526) |  | 0.211 (0.149, 0.256) |  |
| **MTA score** |  | 0.0198 |  | 0.0417 |  | 0.0606 |
| **0** | 1.572 (1.308, 1.918) |  | 0.294 (0.266, 0.334) |  | 0.129 (0.089, 0.156) |  |
| **1** | 1.652 (1.441, 1.934) |  | 0.313 (0.254, 0.380) |  | 0.151 (0.121, 0.201) |  |
| **2** | 1.742 (1.496, 2.142) |  | 0.374 (0.279, 0.460) |  | 0.176 (0.118, 0.227) |  |
| **3** | 2.061 (1.549, 2.418) |  | 0.418 (0.349, 0.566) |  | 0.211 (0.149, 0.256) |  |
| **4** | 2.157 (1.980, 3.550) |  | 0.439 (0.332, 0.560) |  | 0.275 (0.203, 0.346) |  |
| **PA score** |  | 0.5506 |  | 0.5099 |  | 0.3158 |
| **0** | 1.737 (1.432, 2.095) |  | 0.337 (0.258, 0.436) |  | 0.157 (0.124, 0.231) |  |
| **1** | 1.701 (1.472, 1.992) |  | 0.313 (0.268, 0.480) |  | 0.175 (0.138, 0.239) |  |
| **2** | 1.868 (1.649, 2.326) |  | 0.347 (0.275, 0.425) |  | 0.193 (0.127, 0.202) |  |
| **3** | 1.535 (1.485, 2.319) |  | 0.472 (0.346, 0.528) |  | 0.310 (0.161, 0.464) |  |
| Non-normally distributed data are presented as median (IQR); group comparisons used the nonparametric test; *P* < 0.05 was considered statistically significant.  Abbreviations: CSVD, cerebral small vessel disease; CPV, choroid plexus volume; LV, lateral ventricle; 3V, third ventricle; 4V, fourth ventricle; WMH, white matter hyperintensity; BG-EPVS, basal ganglia-enlarged perivascular spaces; CMBs, cerebral microbleeds; BA, brain atrophy; OF, orbito-frontal; AC, anterior cingulate; AT, anterior temporal; FI, fronto-insula; MTA, medial temporal atrophy; PA, posterior atrophy; IQR, inter-quartile range. | | | | | | |

| **Table S7 Correlation between inflammation and conventional CSVD imaging markers in CSVD.** | | | | | | | |
| --- | --- | --- | --- | --- | --- | --- | --- |
|  |  | **WMH**  **score** | **Lacunes**  **score** | **BG-EPVS**  **score** | **CMBs**  **score** | **Total CSVD**  **score** | **Total BA**  **score** |
| **LV CP ∆SIR** | r | 0.509 | 0.294 | -0.206 | 0.004 | 0.235 | 0.096 |
|  | *P* | 0.015 | 0.185 | 0.357 | 0.986 | 0.291 | 0.669 |
| **3V CP ∆SIR** | r | 0.243 | 0.001 | -0.129 | 0.141 | 0.135 | 0.237 |
|  | *P* | 0.275 | 0.997 | 0.566 | 0.533 | 0.550 | 0.288 |
| **4V CP ∆SIR** | r | 0.578 | 0.458 | -0.320 | 0.382 | 0.442 | 0.212 |
|  | *P* | 0.005 | 0.032 | 0.147 | 0.080 | 0.039 | 0.343 |
| **Hip ∆SIR** | r | 0.319 | 0.265 | -0.214 | 0.069 | 0.175 | 0.141 |
|  | *P* | 0.148 | 0.234 | 0.340 | 0.760 | 0.437 | 0.532 |
| **CNh ∆SIR** | r | 0.215 | 0.136 | -0.300 | -0.029 | 0.036 | 0.255 |
|  | *P* | 0.336 | 0.546 | 0.175 | 0.897 | 0.875 | 0.253 |
| **Put ∆SIR** | r | 0.469 | 0.331 | -0.214 | 0.219 | 0.320 | 0.204 |
|  | *P* | 0.028 | 0.133 | 0.339 | 0.327 | 0.146 | 0.362 |
| **GPe ∆SIR** | r | 0.321 | 0.209 | -0.233 | 0.025 | 0.138 | 0.370 |
|  | *P* | 0.145 | 0.352 | 0.296 | 0.911 | 0.539 | 0.090 |
| **Tha ∆SIR** | r | 0.474 | 0.243 | -0.410 | 0.150 | 0.223 | 0.037 |
|  | *P* | 0.026 | 0.276 | 0.058 | 0.505 | 0.318 | 0.872 |
| **Pons ∆SIR** | r | 0.498 | 0.149 | -0.436 | 0.283 | 0.265 | 0.376 |
|  | *P* | 0.018 | 0.508 | 0.043 | 0.202 | 0.234 | 0.084 |
| **MO ∆SIR** | r | 0.438 | 0.317 | -0.323 | 0.133 | 0.239 | 0.310 |
|  | *P* | 0.041 | 0.151 | 0.142 | 0.556 | 0.285 | 0.160 |
| The relationship was analyzed using Pearson correlation; *P* < 0.05 was considered statistically significant.  Abbreviations: CSVD, cerebral small vessel disease; CP, choroid plexus; LV, lateral ventricle; 3V, third ventricle; 4V, fourth ventricle; SIR, signal intensity ratio; WMH, white matter hyperintensity; BG-EPVS, basal ganglia-enlarged perivascular spaces; CMBs, cerebral microbleeds; BA, brain atrophy; Hip, hippocampus; CNh, caudate nucleus head; GPe, globus pallidus externus; Put, putamen; Tha, thalamus; MO, medulla oblongata. | | | | | | | |

| **Table S8 Correlation of inflammation between choroid plexus and deep gray matter nuclei.** | | | | |
| --- | --- | --- | --- | --- |
|  |  | **LV CP ∆SIR** | **3V CP ∆SIR** | **4V CP ∆SIR** |
| **Hip ∆SIR** | r | 0.579 | 0.411 | 0.618 |
|  | *P* | 0.001 | 0.024 | <0.001 |
| **CNh ∆SIR** | r | 0.457 | 0.112 | 0.372 |
|  | *P* | 0.011 | 0.557 | 0.043 |
| **Put ∆SIR** | r | 0.380 | 0.148 | 0.381 |
|  | *P* | 0.039 | 0.435 | 0.038 |
| **GPe ∆SIR** | r | 0.424 | -0.024 | 0.489 |
|  | *P* | 0.019 | 0.899 | 0.006 |
| **Tha ∆SIR** | r | 0.680 | 0.332 | 0.556 |
|  | *P* | <0.001 | 0.073 | 0.001 |
| **Pons ∆SIR** | r | 0.353 | 0.353 | 0.475 |
|  | *P* | 0.056 | 0.056 | 0.008 |
| **MO ∆SIR** | r | 0.284 | 0.443 | 0.501 |
|  | *P* | 0.128 | 0.014 | 0.005 |
| The relationship was analysed using a partial correlation analysis that was adjusted for age and sex.; *P* < 0.05 was considered statistically significant. Abbreviations: CP, choroid plexus; LV, lateral ventricle; 3V, third ventricle; 4V, fourth ventricle; SIR, signal intensity ratio; Hip, hippocampus; CNh, caudate nucleus head; GPe, globus pallidus externus; Put, putamen; Tha, thalamus; MO, medulla oblongata. | | | | |
